# Antenatal Influenza A-Specific IgA, IgM, and IgG Antibodies in Mother’s Own Breast Milk and Donor Breast Milk, and Gastric Contents and Stools from Preterm Infants

**DOI:** 10.3390/nu11071567

**Published:** 2019-07-11

**Authors:** Veronique Demers-Mathieu, Robert K. Huston, Andi M. Markell, Elizabeth A. McCulley, Rachel L. Martin, David C. Dallas

**Affiliations:** 1 Nutrition Program, School of Biological and Population Health Sciences, College of Public Health and Human Sciences, Oregon State University, Corvallis, OR 97331, USA; 2 Department of Pediatrics, Randall Children’s Hospital at Legacy Emanuel, Portland, OR 97227, USA

**Keywords:** passive immunization, maternal immunoglobulins, lactation, prematurity, flu vaccine, human milk

## Abstract

Antenatal milk anti-influenza antibodies may provide additional protection to newborns until they are able to produce their own antibodies. To evaluate the relative abundance of milk, we studied the antibodies specific to influenza A in feeds and gastric contents and stools from preterm infants fed mother’s own breast milk (MBM) and donor breast milk (DBM). Feed (MBM or DBM) and gastric contents (MBM or DBM at 1 h post-ingestion) and stool samples (MBM/DBM at 24 h post-ingestion) were collected, respectively, from 20 preterm (26–36 weeks gestational age) mother-infant pairs at 8–9 days and 21–22 days of postnatal age. Samples were analyzed via ELISA for anti-H1N1 hemagglutinin (anti-H1N1 HA) and anti-H3N2 neuraminidase (anti-H3N2 NA) specificity across immunoglobulin A (IgA), immunoglobulin M (IgM), and immunoglobulin G (IgG) isotypes. The relative abundance of influenza A-specific IgA in feeds and gastric contents were higher in MBM than DBM at 8–9 days of postnatal age but did not differ at 21–22 days. Anti-influenza A-specific IgM was higher in MBM than in DBM at both postnatal times in feed and gastric samples. At both postnatal times, anti-influenza A-specific IgG was higher in MBM than DBM but did not differ in gastric contents. Gastric digestion reduced anti-H3N2 NA IgG from MBM at 21–22 days and from DBM at 8–9 days of lactation, whereas other anti-influenza A antibodies were not digested at either postnatal times. Supplementation of anti-influenza A-specific antibodies in DBM may help reduce the risk of influenza virus infection. However, the effective antibody dose required to induce a significant protective effect remains unknown.

## 1. Introduction

Infants are susceptible to influenza infections and cannot be vaccinated before six months of age [1,2]. Therefore, the Centers for Disease Control and Prevention recommends that all pregnant women receive the inactivated influenza virus vaccine during the second or third trimester [3]. Antenatal vaccination helps protect the infant against influenza infection [4,5,6,7]. Part of that protection stems from the vaccine-stimulated increased maternal blood influenza-specific immunoglobulin G (IgG), which allows increased transfer of influenza-specific IgG across the placenta and leads to increased persistence of these antibodies in the infant bloodstream post-birth [8]. In addition to the changes in blood, antenatal vaccination increases secretion of influenza-specific antibodies into breast milk [9]. Milk anti-influenza antibodies may provide additional protection to newborns until they are able to produce antibodies against the virus [9]. Regardless of whether a mother was recently vaccinated, their breast milk typically contains some influenza-specific Ig as they were likely exposed to the virus or vaccinated in the past [10]. These milk antibodies may be protective against influenza infection [9].

The site of action for milk antibodies is mainly thought to be within the gastrointestinal tract, whereas influenza A infects within the lung mucosal tissue. Thus, the relevance of human milk antibodies to influenza protection in unclear. However, milk influenza A-specific antibodies can be studied as a model for other milk antibodies to enteric pathogens for which the gut is their site of action. To neutralize enteric pathogens throughout the infant gastrointestinal tract, milk antibodies must survive the digestive protease actions. No oral supplementation studies have determined the percentage of remaining relative abundance of anti-influenza A IgA, IgM, and IgG during infant digestion.

In neonatal intensive care units (NICU), very preterm infants are often fed donor breast milk (DBM) to supplement an insufficient maternal supply of mother’s own breast milk (MBM) [11,12]. Whether MBM and DBM differ in milk anti-influenza A antibody relative abundance is unknown. DBM typical processing includes pooling milks from different mothers, pasteurizing (Holder pasteurization, 62.5 °C for 30 min) and freezing and thawing at least twice, which could reduce the antibody abundance. Our previous study demonstrated that total IgA, secretory IgA (SIgA), total IgM, and IgG concentrations were higher in MBM than DBM and higher in gastric contents (1 h post-ingestion) when preterm infants were fed with MBM than when infants were fed DBM [13]. A previous study demonstrated that IgA survived intact after Holder pasteurization in non-centrifuged MBM (0% loss) whereas IgG concentration was reduced (34.3% loss) (IgM was not measured) [14]. In another study, IgA from centrifuged MBM was reduced 22% after Holder pasteurization whereas IgM concentration was completely destroyed [15].

The remaining relative abundance of milk antibodies specific to influenza A virus from MBM or DBM during gastric digestion is unknown and a comparison of mother’s milk and donor milk have not been determined. The aim of this study was to determine the difference of antenatal influenza A-specific IgA, IgM, and IgG antibodies in MBM and DBM, and gastric contents and stools from preterm infants. Milk anti-influenza A neutralizing antibodies can be specific to subtypes of HA or NA [3]. In this study, we examined anti-H1N1 HA and anti-H3N2 NA antibodies.

## 2. Materials and Methods

### 2.1. Participants and Sample Collection

#### 2.1.1. Participants and Enrollment

This study was approved by the Institutional Review Boards of The Legacy Health and Oregon State University (1402–2016, first approved on 05/03/17). Samples were collected from 20 premature-delivering mother-infant pairs ranging in gestational age (GA) at birth from 26 weeks to 36 weeks (Table S1) in the NICU. Parents of all eligible infants in the NICU were approached for participation and informed consent. Clinical data were collected for each mother-infant pair, including GA and postnatal age for both type of feeding (see Table S1). Eligibility criteria included having an indwelling naso/orogastric feeding tube, bolus feeding (<60 min infusion tolerated), feeding volumes of at least four milliliter, and mothers who could produce a volume of MBM adequate for one full-volume feed per day. Exclusion criteria included neonates with diagnoses that were incompatible with life, gastrointestinal system anomalies, major gastrointestinal surgery, severe genitourinary anomalies and significant metabolic, or endocrine diseases.

#### 2.1.2. Feeding and Sampling

**Figure 1:**
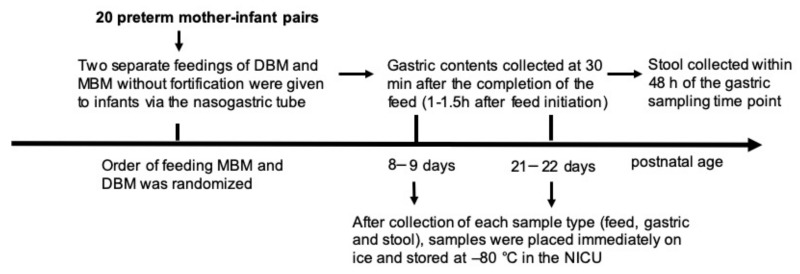
Schema of study design to determine the difference of antenatal influenza-specific antibodies in mother’s breast milk (MBM) and donor breast milk (DBM) in gastric contents and stool samples from preterm infants.

To compare the effects of DBM and MBM, two separate feedings of DBM and MBM without fortification were given to infants. Milk and gastric samples (2 mL) were collected on 8–9 and 21–22 days of postnatal age (Figure 1). At both sample time periods, each infant received two of the normal eight daily feedings as unfortified MBM or DBM on alternate days (randomized order). The order of feeding MBM and DBM was randomized to control for any potential effect of infant day of life on antibody digestion. The pool of DBM was acquired from two batches at Northwest Mother’s Milk Bank. Three-liter batches were pasteurized and frozen in 50 mL doses so that only a small fraction was thawed for each infant feeding. The power analysis based in our previous study indicated that at least 15 infants were required to compare DBM and MBM-fed infants [16].

The protocol of feeding is described in our previous study [13]. Milk (either MBM or DBM) was fed to the infant via the nasogastric tube with a feeding pump set to deliver the entire bolus over 30–60 min. A sample of the gastric fluid was collected 30 min after the completion of the feed (1–1.5 h after feed initiation). Stool (1 g) was collected within 48 h of the gastric sampling time point and was recovered from the diaper and scraped into a sterile jar. Stool sample collection was not specific to DBM/MBM and thus represents stools deriving from a mixture of DBM and MBM feeding. After collection of each sample type (feed, gastric and stool), samples were placed immediately on ice and stored at −80 °C in the NICU. Samples were transported on dry ice to the Dallas laboratory at Oregon State University for sample analysis.

### 2.2. Sample Preparation and ELISAs

Feed (MBM and DBM) and gastric samples were thawed at 4 °C, pH was determined and samples (1 mL) were centrifuged at 3500× *g* for 30 min at 4 °C. The infranate was collected, separated into aliquots (100 μL) and stored at −80 °C. Frozen stool samples (0.1 g) were diluted in 700 μL of phosphate-buffered saline pH 7.4 (Thermo Fisher Scientific, MA, USA) with 0.05% Tween-20 (Bio-Rad Laboratories, Irvine, CA, USA) (PBST) and 3% fraction V bovine serum albumin solution (Innovative Research, Novi, MI, USA). Diluted stool samples were mixed by vortex for 2 min and the vials were centrifuged at 3500× *g* for 20 min at 4 °C. The supernatant was collected, separated into aliquots (100 μL) and stored at −80 °C. Sample pH and protein concentration measurements were performed and results were published previously [13].

The spectrophotometric ELISAs were recorded with a microplate reader (Spectramax M2, Molecular Devices, Sunnyvale, CA, USA) with two replicates of blanks, standards and samples. Clear flat-bottom 96-well plates (Thermo Fisher Scientific, Waltham, MA, USA) were coated with 0.75 μg/mL (100 μL) of influenza A/Hong Kong/4801/2014 H3N2 neuraminidase (H3N2 NA) or influenza A/California/07/2009 H1N1 hemagglutinin (H1N1 HA) (Sino Biological US Inc., Wayne, PA, USA). These antigens were chosen based on the strains present in the vaccine (Flucelvax Quadrivalent, 2017–2018 formula, Seqirus, Holly Springs, NC, USA, Table S2) that mothers received for 2017–2018 when samples were collected. Plates were incubated overnight at 4 °C. After incubation, plates were washed three times with PBST. Blocking buffer (100 μL PBST with 3% fraction V bovine serum albumin solution) was added in all wells and incubated for 1 h at room temperature. Standards were prepared using human serum from a female adult that received the influenza vaccine (Flucelvax Quadrivalent, 2017–2018 formula, Seqirus, Holly Springs, NC, USA, Table S2) four months prior. Blood was collected into six BD vacutainer serum separation tubes (SSTTM) (Becton Dickinson, Franklin Lakes, NJ, USA), incubated at room temperature for 5 min, and centrifuged at 3900× *g* for 5 min. Serum was aliquoted into several vials and stored at −80 °C until used. The relative abundance of influenza A antibodies was derived by interpolation from the standard curve generated from human serum with the assigned quantity of anti-influenza A antibodies expressed in ELISA units/mL (EU/mL) (non-diluted serum (1x) = 50,000 arbitrary EU/mL). The standard curves were prepared using a dilution series of standard human serum in blocking buffer and the final concentration covered a range from 12.5× (4000 EU/mL) to 25,600× (1.95 EU/mL). Feed (MBM or DBM) and gastric samples were diluted 10× with blocking buffer for anti-influenza IgA, IgM, and IgG measurements whereas a 2× dilution was used for the prediluted stool samples. For each step (addition of 100 μL standards/samples and secondary antibodies at 1 μg/mL), washing and incubation for 1 h at room temperature were performed. The detection antibodies were goat anti-human IgA alpha-chain: horseradish peroxidase (HRP) for anti-influenza IgA, goat anti-human IgM mu-chain:HRP for anti-influenza IgM and goat anti-human IgG gamma-chain:HRP for anti-influenza IgG (Bio-Rad Laboratories). The substrate 3, 3′, 5, 5′-tetramethylbenzidine (Thermo Fisher Scientific) (1×, 100 μL) was added for 5 min at room temperature followed by addition of 2 N sulfuric acid (100 μL) to stop the reaction. Optical density was measured at 450 nm.

### 2.3. Pasteurization Effect on Antibodies Specific to Influenza A

To examine the effects of pasteurization directly, a breast milk sample was collected from one mother who was vaccinated with the 2017–2018 influenza vaccine at 25 weeks of pregnancy and gave birth at term. At 12 days of lactation, the mother pumped and collected 150 mL of milk into a sterile plastic bag. Six 1 mL aliquots were centrifuged at 4000× *g* for 30 min, 4 °C and infranates were collected (skim). Three of these aliquots were used for the control raw human milk (RHM) and 3 others were pasteurized at 62.5 °C for 30 min, referred to as the pasteurized skimmed human milk (PSHM). Three other 1-mL aliquots were pasteurized whole (referred to as pasteurized whole human milk, PWHM) and centrifuged at 4000× *g* for 30 min at 4 °C prior to ELISA.

### 2.4. Statistical Analyses

Wilcoxon matched-pairs signed-rank test in GraphPad Prism software (version 8) was used to compare relative abundance of each anti-influenza antibody isotype (EU/mL) in milk and in gastric contents between MBM and DBM (type of feeding) within the same mother-infant pairs at 8–9 days and 21–22 days of postnatal age. Student’s t-tests were used to evaluate the effect of whether or not infants received antibiotics in gastric and stool samples from both feeding types (MBM and DBM) (Table S3). One-way ANOVA followed by Dunnett’s multiple comparisons test was performed to compare RHM to PSHM and PWHM. Differences were designated significant at *p* < 0.05. Geometric mean relative abundances (GMRA) of these antibodies in samples at 8–9 days and 21–22 days of postnatal age were also calculated.

## 3. Results

### 3.1. Infant Demographics

Demographic details for the preterm-delivering mother-infant pairs are presented in Table S1.

### 3.2. Anti-Influenza Antibody Relative Abundance in Feeds

**Figure 2:**
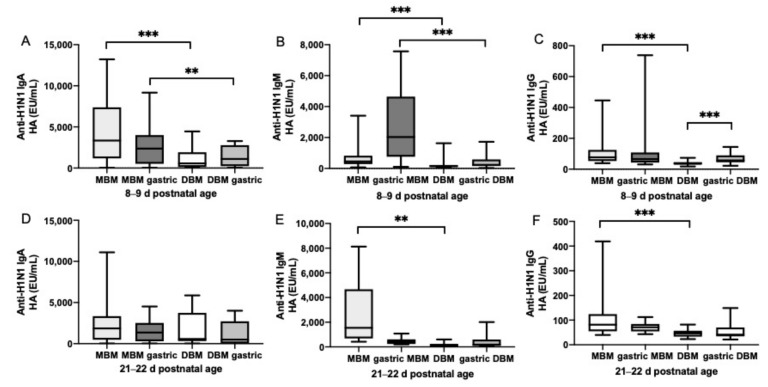
Milk antibodies specific to anti-H1N1 hemagglutinin (anti-H1N1 HA) in mother’s own breast milk (MBM) and donor breast milk (DBM) feeds, and gastric samples from preterm infants fed MBM and DBM (26–36 wk of GA). Relative abundance of (**A**) anti-H1N1 HA IgA, (**B**) anti-H1N1 HA IgM, and (**C**) anti-H1N1 HA IgG at 8–9 days of postnatal age. Relative abundance (EU/mL) of (**D**) anti-H1N1 HA IgA, (**E**) anti-H1N1 HA IgM, and (**F**) anti-H1N1 HA IgG at 21–22 days of postnatal age. Values are min, max, and median, *n* = 20 for 8–9 days and *n* = 16 for 21–22 days of postnatal age. Asterisks show statistically significant differences between variables (*** *p* < 0.001; ** *p* < 0.01) using the Wilcoxon matched-pairs signed-rank test.

The relative abundance of H1N1 HA- (Figure 2) and H3N2 NA-specific IgA (Figure 2) were 3.6- and 2-fold higher, respectively, in MBM than DBM feeds given at 8–9 days of postnatal age but did not differ at 21–22 days. H1N1 HA- (10- and 3-fold) and H3N2 NA-specific IgM (13- and 8-fold) were higher in MBM than in DBM feeds given at 8–9 days and 21–22 days of postnatal age, respectively. H1N1 HA-specific IgG was 2-fold higher in MBM than DBM feeds given at 8–9 days and 21–22 days of postnatal age. H3N2 NA-specific IgG was 30% higher in MBM than DBM feeds given at 8–9 days but did not differ at 21–22 days. The GMRA of each anti-influenza isotype are summarized in Table S4.

### 3.3. Anti-Influenza Antibody Relative Abundance in Gastric Contents

At 8–9 days of postnatal age, relative abundance of H1N1 HA- (Figure 2) and H3N2 NA-specific IgA (Figure 2) were 4- and 7-fold higher in gastric contents from infants fed MBM than those fed DBM. At 21–22 days, H3N2 NA-specific IgA was 4-fold higher in gastric contents after feeding MBM than after feeding DBM but did not differ for H1N1 HA-specific IgA. H1N1 HA- and H3N2 NA-specific IgM were 6- and 11-fold higher in gastric contents from infants fed MBM than those fed DBM at 8–9 days. At 21–22 days, H3N2 NA-specific IgM was 5-fold higher in MBM than DBM but did not differ for H1N1 HA-specific IgM. H1N1 HA- and H3N2 NA-specific IgG in gastric contents did not differ after feeding infants MBM and those fed DBM. The GMRA of each anti-influenza isotype are summarized in Table S5.

### 3.4. Gastric Digestion of Anti-Influenza Antibody from MBM and DBM

**Figure 3:**
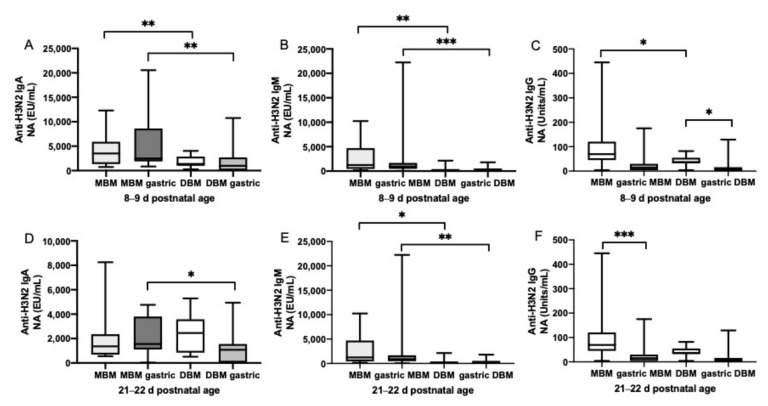
Milk antibodies specific to anti-H3N2 neuraminidase (anti-H3N2 NA) in mother’s own breast milk (MBM) and donor breast milk (DBM) feeds, and gastric samples from preterm infants fed MBM and DBM (26–36 wk of GA). Relative abundance of (**A**) anti-H3N2 NA IgA, (**B**) anti-H3N2 NA IgM, and (**C**) anti-H3N2 NA IgG at 8–9 d postnatal age. Relative abundance (EU/mL) of (**D**) anti-H3N2 NA IgA, (**E**) anti-H3N2 NA IgM, and (**F**) anti-H3N2 NA IgG at 21–22 days of postnatal age. Values are min, max, and median, *n* = 20 for 8–9 days and *n* = 16 for 21–22 days of postnatal age. Asterisks show statistically significant differences between variables (*** *p* < 0.001; ** *p* < 0.01; * *p* < 0.05) using the Wilcoxon matched-pairs signed-rank test.

Relative abundance of H1N1 HA- (Figure 1) and H3N2 NA-specific IgA and IgM (Figure 3) did not significantly decrease from feed to gastric contents in infants fed MBM or DBM at 8–9 days or 21–22 days of postnatal age. H1N1 HA-specific IgG from MBM did not change from feed to gastric contents at 8–9 days or 21–22 days, whereas H1N1 HA-specific IgG from DBM slightly increased in gastric contents at 8–9 days but did not differ at 21–22 days of postnatal age. H3N2 NA-specific IgG from DBM decreased in the gastric contents at 8–9 days but did not differ at 21–22 days. H3N2 NA-specific IgG from MBM decreased in gastric contents at 21–22 days but not at 8–9 days. The GMRA abundances of each anti-influenza isotype are summarized in Table S6.

### 3.5. Postnatal Age Effects on Anti-Influenza A Antibodies in Preterm Infant Stools Derived from Mixed MBM/DBM

The relative abundance of H1N1 HA- and H3N2 NA-specific IgA, IgM and IgG in infant stools did not differ between 8–9 days and 21–22 days of postnatal age (Table S7).

### 3.6. Pasteurization Effects on Human Milk Anti-Influenza A Antibodies

**Figure 4:**
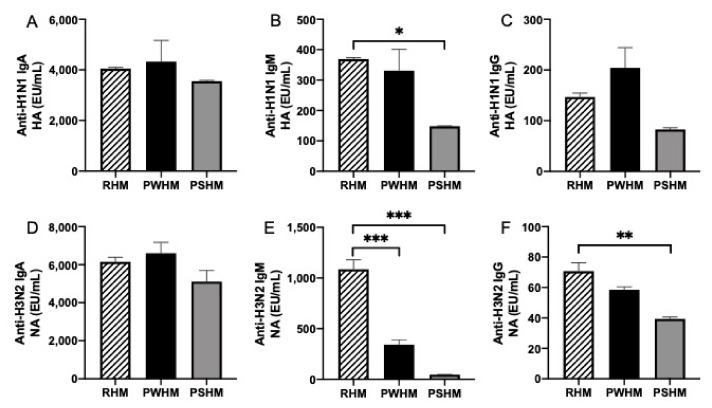
Comparison of immunoglobulin relative abundance between raw human milk (RHM) and pasteurized whole human milk (PWHM) or pasteurized skimmed human milk (PSHM) (*n* = 3). Relative abundance of anti-H1N1 HA (**A**) IgA, (**B**) IgM, (**C**) IgG, and of anti-H3N2 NA. (**D**) IgA, (**E**) IgM, and (**F**) IgG in mother’s milk. Milk samples were from one mother who delivered one term infant at 38 wk of GA and collected milk at 12 days of postnatal age. Asterisks show statistically significant differences between variables (*** *p* < 0.001; ** *p* < 0.01; * *p* < 0.05) using one-way ANOVA followed by Dunnett’s multiple comparisons test.

The anti-influenza antibodies in RHM to PWHM and PSHM were compared to determine the effect of Holder pasteurization on them. Whole and skim breast milk were pasteurized because previous literature reported different results with and without delipidation before pasteurization [14,15]. The relative abundance of anti-H1N1 HA IgA, anti-H3N2 NA IgA, and anti-H1N1 HA IgG did not differ between RHM and PWHM or PSHM (Figure 4). Anti-H1N1 HA IgM, anti-H3N2 NA IgM, and anti-H3N2 NA IgG in RHM were 2.5-, 23-, and 2-fold higher than in PSHM. Anti-H3N2 NA IgM in RHM was 3-fold higher than in PWHM. Anti-H1N1 HA IgM, anti-H3N2 NA IgM, and anti-H3N2 NA IgG in PWHM were 2.5-, 7.3-, and 1.5-fold higher than PSHM but did not differ for other antibodies.

## 4. Discussion

This study aimed to determine whether the relative abundance of maternal milk anti-influenza A antibodies differed between MBM and DBM and across their digestion in gastric contents and in stool of preterm infants. Influenza A viruses vary based on their hemagglutinin (HA) and neuraminidase (NA) subtypes; each influenza A virus has a specific combination of an HA and an NA subtype [3]. Milk anti-influenza A neutralizing antibodies can be specific to subtypes of HA or NA. In this study, we examined anti-H1N1 HA and anti-H3N2 NA antibodies. Anti-influenza IgA level was previously evaluated in human milk via ELISA [9]. The anti-H1N1 HA IgA relative abundance measured herein was higher than those measured previously in term-delivering influenza-vaccinated mothers by Schlaudecker et al. [9]. However, abundance values of milk antibodies cannot be compared with those reported in other studies because different human serums were used to prepare the standard curve for each anti-influenza antibody isotype. No commercial standard exists for anti-influenza IgA, IgM, and IgG; therefore, determining absolute concentrations was not possible in this study. This research is the first to determine the anti-influenza A IgM and IgG relative abundance in breast milk.

Anti-influenza A H1N1 HA and H3N2 NA IgA in MBM were higher than in DBM during the first week of postnatal age for both feeds and gastric contents from preterm infants but did not differ during the third week for either feed or gastric contents. Neither anti-influenza A H1N1 HA nor H3N2 NA IgA was reduced by pasteurization of whole or skim breast milk. Therefore, we speculate that high relative abundance of anti-influenza IgA in MBM is related to early GA and early postnatal age of infants when mothers delivered prematurely. DBM is donated from mothers delivering mostly term infants and milk is usually collected later than three months of lactation, which could have different milk antibody levels compared with those from preterm-delivering mothers at early lactation times. Our recent clinical study demonstrated that antibody concentrations (total IgA and SIgA) were higher in MBM than DBM in feeds and gastric samples (1 h post-feeding) from preterm infants [13]. A previous study found that IgA concentration in preterm milk was higher than in term milk from 3 to 15 days of lactation time [17]. Total IgA concentration in colostrum from preterm-delivering mothers was higher than colostrum (1–3 days of lactation time) from term-delivering mothers in several studies [17,18,19]. However, our previous study showed that total IgA did not differ between preterm milk and term milk from 6 days to 28 days of lactation time [16]. No study has previously evaluated the difference in relative abundance of milk anti-influenza IgA antibodies between preterm- and term-delivering mothers or between early and late lactation time. Our results suggest that the provision of passive immune protection with milk anti-influenza A IgA was highest when infants are fed MBM during their first week of postnatal age.

Anti-influenza A H1N1 HA and H3N2 NA IgM in MBM were higher than in DBM during the first and third week of postnatal age in both feeds and in infant gastric samples. Among isotypes, anti-influenza IgM relative abundance differed most between DBM and MBM. Anti-H1N1 HA IgM was reduced when skim milk was pasteurized but not when whole breast milk was pasteurized. On the other hand, anti-H3N2 NA IgM was decreased by pasteurizing whole and skim milk. We hypothesize that the lower relative abundance of anti-influenza A IgM in DBM compared with MBM was due to the pasteurization of pooled donor milk after fat loss during freezing and thawing. The reduction of anti-influenza IgM was not likely due to the difference of GA or lactation time between MBM (preterm-delivering mothers) and DBM (term-delivering mothers). Anti-influenza IgM did not change across lactation time (1 weeks to 3 weeks) in milk from preterm-delivering mothers. Our recent clinical study demonstrated that total IgM concentration was higher in MBM than DBM in feeds and gastric samples (1 h post-feeding) from preterm infants [13]. Previous studies showed that IgM concentration in preterm-delivering mothers was lower than [16], higher than [17] or similar to [20,21] term-delivering mothers, suggesting a high variability of IgM concentration between mothers. A strong variation of anti-H3N2 NA IgM in MBM from preterm-delivering mothers (0 EU/mL to 4301 EU/mL at 1 week; 0–900 EU/mL at 3 weeks) was observed, which could be due to the time of vaccination during pregnancy and the mother’s immune response to the vaccine.

Anti-H1N1 HA IgG was higher in MBM than in DBM fed during the first and third week of postnatal age. Pasteurization did not affect anti-H1N1 HA IgG in whole or skim breast milk. The higher relative abundance of anti-H1N1 HA IgG in MBM could be due to the difference of GA and lactation time between preterm-delivering mothers and term-delivering mothers (DBM). Anti-influenza A H1N1 HA and H3N2 NA IgG did not differ in gastric contents of infants fed MBM and DBM either at 1 week or 3 weeks of postnatal age. Interestingly, anti-H3N2 NA IgG was higher in MBM than in DBM during the first week but did not differ at third week of MBM lactation. Anti-H3N2 NA IgG relative abundance in skimmed milk but not whole milk was reduced by pasteurization. We speculate that anti-H3N2 NA IgG was low in DBM due to the time of lactation. Our recent clinical study demonstrated that total IgG concentration was higher in MBM than DBM in feed and gastric samples (1 h post-feeding) from preterm infants [13].

Gastric digestion reduced anti-H3N2 NA IgG from MBM at 21–22 days and from DBM at 8–9 days of postnatal age, but the other anti-influenza A antibodies were not digested in the gastric contents of preterm infants. A slight increase of anti-H3N2 NA IgG from DBM in gastric contents at 8–9 days of postnatal age was observed, which could be due to contamination of the gastric contents with residual from previous feeds based on MBM.

All influenza A antibodies most likely resisted preterm infant overall digestion to some extent as all were detected in stools 24 h post-feeding. Antibodies detected in stool samples could derive from the MBM and/or DBM feeding or potentially be generated by the infant. Anti-H1N1 and anti-H3N2 antibodies in stool samples did not differ between the first and third week of postnatal age.

Five infants among twenty received antibiotics (Table S1). Provision of antibiotics could change the microbiome and alter the survival of maternal influenza antibodies to the stool. However, no differences were detected in their survival in the gastric contents and stools between infants with and without antibiotics (Table S3).

Schlaudecker et al. [9] demonstrated that anti-influenza IgA level in breast milk correlated inversely with frequency of respiratory illness with fever in term infants. The authors hypothesized that orally ingested milk antibodies may contribute to immune protection within airways against respiratory infections [9]. However, this high protection effect could also be due to the high anti-influenza antibody concentration (IgG) in mother’s blood that was transmitted to the infant across the placenta.

The function of milk anti-influenza antibodies may be to neutralize viruses in breast milk or in the gastrointestinal or respiratory tracts. Small amounts of breast milk can reach the infant respiratory tract as a result of regurgitation and inhalation of breast milk during and after feeding [22]. Therefore, milk influenza-specific antibodies may reach the respiratory tract through regurgitation and inhalation of breast milk and protect the mucosa of the respiratory tract against influenza A viruses, as previously hypothesized for milk anti-respiratory syncytial virus-specific Ig [23]. Nutritional intervention studies are needed to confirm whether the milk antibodies specific to influenza A can contribute to protection against respiratory pathogens.

Milk antibodies may also interact with immune cells within the infant intestinal lumen to protect against pathogens. Maternal milk-derived SIgA2 and IgA2 (but not IgA1, IgG or IgM) can adhere to the apical surfaces of mice intestinal M cells and be transported across the epithelial cell layer [24]. IgA1 has a heavily O-glycosylated hinge region, whereas the smaller IgA2 hinge region is not glycosylated. The investigators speculated that the extended hinge of IgA1 may interfere with binding to the M cell receptor [24]. A hypothetical IgA receptor on M cells could mediate the delivery of SIgA2 from the intestinal lumen to the mucosa-associated lymphoid tissues. SIgA2-antigen complexes absorbed across M cells could be sampled by subepithelial B lymphocytes and dendritic cells [25]. Indeed, SIgA was detected on the apical surface of M cells in the infant ileum, suggesting that they may be able to endocytose SIgA. Therefore, milk anti-influenza antibodies complexed with influenza antigens could hypothetically be absorbed by M cells, sampled by leukocytes and lead to altered mucosal immune response in the respiratory tract.

A limitation in our study was the absence of information on vaccination (vaccinated or not and dates) from recruited mothers in order to demonstrate a positive correlation between vaccinated mothers and anti-influenza antibody level in breast milk. However, this association was already demonstrated in previous a study for anti-influenza IgA in breast milk [9].

Another study limitation is that too few subjects were included to detect many differences between MBM and DBM feeds and gastric contents when grouped by GA (26–27 weeks, 30–31 weeks, and 35–36 weeks).

## 5. Conclusions

The present study suggests that passive immunization with milk anti-influenza A IgA may have been highest when infants were fed MBM rather than DBM during the first week of postnatal age. Anti-influenza A IgM was higher in MBM than in DBM during the first and third week of postnatal age in both type of samples (feeds and gastric samples). Anti-influenza IgM differed most between DBM and MBM, likely due to its sensitivity to pasteurization. Anti-influenza IgG relative abundance was higher in MBM than DBM feeds but did not differ in gastric contents at first and third week of postnatal age. Passive immune protection with maternal anti-influenza antibodies from MBM may provide infant protection. Milk antibodies could neutralize viruses within the mammary gland, in the infant mouth and after entering their respiratory system. Supplementing DBM with additional influenza A-specific antibodies could benefit infants.

## Supplementary Materials

The following are available online at https://www.mdpi.com/2072-6643/11/7/1567/s1, Table S1. Demographics of preterm-delivering mother-infant pairs sampled for MBM and DBM feeds, gastric contents (1-h postprandial time) and stools (24-h post-feeding); Table S2. Description of influenza vaccine (Flucelvax Quadrivalent 2017-2018 Formula) given to mothers during pregnancy; Table S3. Statistical results (*p*-values) for Student’s *t*-tests comparing antibody concentration between infants that received antibiotics (*n* = 5 at 8–9 days and *n* = 5 at 21–22 days of postnatal age) and infants that did not receive antibiotics (*n* = 15 at 8–9 days and *n* = 11 at 21–22 days) in gastric and stool samples. G, gastric contents; S, stool; Table S4. GMRA of anti-influenza A IgA, IgM and IgG (H1N1 HA and H3N2 NA) in MBM and DBM at 8–9 days and for 21–22 days of postnatal age. MBM samples were from 20 preterm-delivering mothers (26–36 wk of GA). *P*-values were calculated using the Wilcoxon matched-pairs signed-rank test; Table S5. GMRA of anti-influenza IgA, IgM, and IgG (H1N1 HA and H3N2 NA) in gastric contents fed MBM and DBM feeds. Gastric contents from infants fed MBM or DBM were from 20 mother-preterm pairs (26–36 wk of GA at 8–9 days and for 21–22 days of postnatal age. *p*-values were calculated using the Wilcoxon matched-pairs signed-rank test; Table S6. *p*-value for the difference between MBM and gastric MBM or between DBM and gastric DBM on anti-influenza IgA, IgM, and IgG reactivities (H1N1 HA and H3N2 NA). Feed and gastric samples were from 20 mother-preterm pairs (26–36 wk of GA). *p*-values were calculated using the Wilcoxon matched-pairs signed-rank test; Table S7. GMRA for anti-influenza IgA, IgM and IgG antibodies (H1N1 HA and H3N2 NA) in stools from preterm infants. Stool samples were from 20 preterm infants at 26–36 wk of GA. *p*-values were calculated using the Wilcoxon matched-pairs signed-rank test between 8–9 days and 21–22 days of postnatal age.
