# Supplementary material for: Antenatal Influenza A-Specific IgA, IgM, and IgG Antibodies in Mother’s Own Breast Milk and Donor Breast Milk, and Gastric Contents and Stools from Preterm Infants
Source: Nutrients. 2019 Jul 11;11(7):1567. doi: 10.3390/nu11071567 (PMC6682892; doi:10.3390/nu11071567)
Supplement: Supplementary file 1 [file nutrients-11-01567-s001.pdf]

Table S1. Demographics of preterm-delivering mother-infant pairs sampled for MBM and DBM feeds, gastric contents (1-h postprandial time) and stools (24-h post-feeding).

Demographics	Preterm-Delivering Mother Infant Pairs [1–2]
GA, week	30 ± 3 (26–36)
Postnatal age for mother's milk, days	8.6 ± 0.1 and 21.4 ± 0.1
Postnatal age for donor breast milk, days	8.4 ± 0.1 and 21.6 ± 0.1
Birth weight at birth, kg	1.5 ± 0.7 (0.7–3.6)
Weight gain velocity, g/kg/day [3]	11 ± 4
Length gain velocity, cm/wk [3]	1.0 ± 0.3
Head circumference gain velocity, cm/wk [3]	0.6 ± 0.3
Volume of feeding at 8–9 days of postnatal age, mL	16 ± 12 (2.5–38)
Volume of feeding at 21–22 days of postnatal age, mL	28 ± 10 (14–49)
Infant sex	10 females; 10 males
Infant of a diabetic mother, *n*	3
SGA10, less than the 10th percentile [4], *n*	2
SGA3, less than the 3rd percentile [4], *n*	1
Intrauterine illicit drug exposure, *n*	2
C-section, *n*	16
Retinopathy of prematurity, *n*	0
Chronic lung disease, *n*	2
Gram-negative sepsis, *n*	1
Gram-positive sepsis or fungal sepsis	0
Late-onset sepsis	1
Antibiotics[5] received postnatally, *n*	5
Necrotizing enterocolitis	0
Gastrointestinal bleeding	0
Cardiopulmonary resuscitation	0
Infant death	0
Length of stay in NICU	47 ± 29 (10–109)

[1] Values are mean ± SD (range); [2] Number of paired milk and gastric and stool samples from preterm infants is n = 20; [3] Birth to discharge; [4] Small for gestational age (SGA) on the Fenton 2013 Growth chart; [5] Antibiotics were ampicillin/cefdinir for 3 infants and ampicillin/gentamycin for 2 infants.

Table S2. Description of influenza vaccine (Flucelvax Quadrivalent 2017–2018 Formula) given to mothers during pregnancy.

Information	Description
Hemagglutinin (HA) dose	60 (15 mcg HA of each strain) mcg per 0.5-mL
Eight influenza strains	A/Singapore/GP1908/2015 IVR-180 (H1N1) (an A/Michigan/45/2015-like virus)
	A/Singapore/GP2050/2015 (H3N2) (an A/Hong Kong/4801/2014-like virus)
	B/Utah/9/2014 (a B/Phuket/3073/2013-like virus)
	B/Hong Kong/259/2010 (a B/Brisbane/60/08-like-virus)
Manufacturer	Seqirus Inc, Holly Springs, NC, USA
Inactivated vaccine	Intramuscular injection in the region of the deltoid muscle of the upper arm.

Table S3. Statistical results (*p*-values) for Student's *t*-tests comparing antibody concentration between infants that received antibiotics (*n* = 5 at 8–9 days and *n* = 5 at 21–22 days of postnatal age) and infants that did not receive antibiotics (*n* = 15 at 8–9 days and *n* = 11 at 21–22 days) in gastric and stool samples. G, gastric contents; S, stool.

Antibodies	G DBM8–9	G DBM21–22	G MBM8–9	G MBM21–22	S 8–9	S 21–22
H1N1 HA IgA	0.23	0.51	0.61	0.12	0.41	0.41
H3N2 NA IgA	0.58	0.23	0.48	0.92	0.46	0.09
H1N1 HA IgM	0.73	0.78	0.27	0.60	0.39	0.35
H3N2 NA IgM	0.25	0.49	0.27	0.71	0.16	0.66
H1N1 HA IgG	0.30	0.43	0.61	0.27	0.75	0.43
H3N2 NA IgG	0.56	0.22	0.28	0.78	0.90	0.36

Table S4. Geometric mean relative abundance (GMRA) of anti-influenza A IgA, IgM and IgG (H1N1 hemagglutinin (H1N1 HA) and H3N2 neuraminidase (H3N2 NA)) in mother's own breast milk (MBM) and donor breast milk (DBM) at 8–9 days and for 21–22 days of postnatal age. MBM samples were from 20 preterm-delivering mothers (26–36 weeks of gestational age). *p*-values were calculated using the Wilcoxon matched-pairs signed-rank test.

Isotype in Feeds	Postnatal Age (days)	Specificity	MBM GMRA (EU/mL, 95% CI)	DBM GMRA (EU/mL, 95% CI)	*p*-Value
IgA	8–9	H1N1 HA	1878 (139–3616)	523 (0–1339)	0.001
IgA	21–22	H1N1 HA	1149 (0–2928)	637.9 (0–1356.1)	0.33
IgA	8–9	H3N2 NA	2936 (1519–4353)	1468 (960–1976)	0.008
IgA	21–22	H3N2 NA	1475 (246–2705)	1899 (1099–2700)	0.89
IgM	8–9	H1N1 HA	1413 (360–2466)	139 (57–221)	<0.001
IgM	21–22	H1N1 HA	457 (0–951)	155 (0–360)	0.002
IgM	8–9	H3N2 NA	1688 (292–3083)	132 (0–357)	0.003
IgM	21–22	H3N2 NA	933 (0–2334)	115 (0–338)	0.015
IgG	8–9	H1N1 HA	89 (51–128)	42.1 (36–48)	<0.001
IgG	21–22	H1N1 HA	85 (33–136)	38 (31–45)	<0.001
IgG	8–9	H3N2 NA	8.7 (0–24)	25 (11–38)	0.031
IgG	21–22	H3N2 NA	15 (3.7–26)	18 (0.6–35)	0.62

Table S5. Geometric mean relative abundance (GMRA) of anti-influenza IgA, IgM and IgG (H1N1 hemagglutinin (H1N1 HA) and H3N2 neuraminidase (H3N2 NA)) in gastric contents fed mother's own breast milk (MBM) and donor breast milk (DBM) feeds. Gastric contents from infants fed MBM or DBM were from 20 mother-preterm pairs (26–36 weeks of gestational age at 8–9 days and for 21–22 days of postnatal age. P-values were calculated using the Wilcoxon matched-pairs signed-rank test.

Isotype in Gastric	Postnatal Age (Days)	Specificity	MBM GMRA (EU/mL, 95% CI)	DBM GMRA (EU/mL, 95% CI)	*p*-Value
IgA	8–9	H1N1 HA	1499 (320–2678)	406 (0–582)	0.034
IgA	21–22	H1N1 HA	699 (109–1289)	549 (0–1359)	0.32
IgA	8–9	H3N2 NA	3536 (844–6229)	542 (0–1807)	0.002
IgA	21–22	H3N2 NA	1493 (730–2256)	414 (0–1185)	0.039
IgM	8–9	H1N1 HA	1531 (438–2623)	267 (72–462)	0.001
IgM	21–22	H1N1 HA	405 (270–541)	201 (0–497)	0.19
IgM	8–9	H3N2 NA	1547 (0–4301)	145 (0–360)	<0.001
IgM	21–22	H3N2 NA	443 (0–903)	88 (0–224)	0.006
IgG	8–9	H1N1 HA	48 (30–66)	60 (47–74)	0.26
IgG	21–22	H1N1 HA	82 (6.0–158)	48 (30–66)	0.20
IgG	8–9	H3N2 NA	17 (0–37)	6.6 (0–19)	0.068
IgG	21–22	H3N2 NA	9.6 (2.3–17)	15 (0–39)	0.22

Table S6. *p*-value for the difference between MBM and gastric MBM or between DBM and gastric DBM on anti-influenza IgA, IgM and IgG reactivities (H1N1 HA and H3N2 NA). Feed and gastric samples were from 20 mother-preterm pairs (26–36 weeks of gestational age). *p*-values were calculated using the Wilcoxon matched-pairs signed-rank test.

Isotype in Feed-Gastric	Postnatal Age (Days)	Specificity (EU/mL)	MBM vs. Gastric MBM (*p*-Value)	DBM vs. Gastric DBM (*p*-Value)
IgA	8–9	H1N1 HA	0.25	0.49
IgA	21–22	H1N1 HA	0.23	0.99
IgA	8–9	H3N2 NA	0.33	0.74
IgA	21–22	H3N2 NA	0.27	0.064
IgM	8–9	H1N1 HA	0.49	0.083
IgM	21–22	H1N1 HA	0.22	0.34
IgM	8–9	H3N2 NA	0.52	0.53
IgM	21–22	H3N2 NA	0.22	0.19
IgG	8–9	H1N1 HA	0.43	0.001
IgG	21–22	H1N1 HA	0.11	0.13
IgG	8–9	H3N2 NA	0.24	0.020
IgG	21–22	H3N2 NA	0.001	0.37

Table S7. GMRA for anti-influenza IgA, IgM and IgG antibodies (H1N1 hemagglutinin (H1N1 HA) and H3N2 neuraminidase (H3N2 NA)) in stools from preterm infants. Stool samples were from 20 preterm infants at 26–36 weeks of gestational age. *p*-values were calculated using the Wilcoxon matched-pairs signed-rank test between 8–9 days and 21–22 days of postnatal age.

Isotype in Stool	Specificity	GMRA 8–9 Days (EU/mL, 95% CI)	GMRA 21–22 Days (EU/mL, 95% CI)	*p*-Values
IgA	H1N1 HA	61.6 (20.4–102.8)	71.2 (41.3–101.1)	0.40
IgA	H3N2 NA	135.3 (72.3–198.4)	125.0 (7.0–243.1)	0.98
IgM	H1N1 HA	59.2 (0–209.2)	46.1 (0–132.9)	0.34
IgM	H3N2 NA	40.1 (0–112.7)	58.3 (10.8–105.8)	0.99
IgG	H1N1 HA	1.3 (0–4.2)	0.7 (0.6–0.9)	0.20

IgG	H3N2 NA	1.1 (0.4–1.8)	1.0 (0.7–1.3)	0.39
